# Cryoneurolysis for the management of chronic pain in patients with knee osteoarthritis; a double-blinded randomized controlled sham trial

**DOI:** 10.1186/s12891-021-04102-1

**Published:** 2021-02-26

**Authors:** Niels-Peter Brøchner Nygaard, Carsten Koch-Jensen, Henrik Bjarke Vægter, Niels Wedderkopp, Morten Blichfeldt-Eckhardt, Bibi Gram

**Affiliations:** 1 Research Unit of Health Science, Hospital of South West Jutland, University Hospital of Southern Denmark, Esbjerg, Denmark; 2 Department of Regional Health Research, University of Southern Denmark, Odense, Denmark; 3 Department of Neurology, Hospital South West Jutland, University Hospital of Southern Denmark, Esbjerg, Denmark; 4 Pain Research Group, Odense University Hospital, Odense, Denmark; 5 Department of Clinical Research, Faculty of Health Sciences, University of Southern Denmark, Odense, Denmark; 6 Department of Orthopedics, Hospital South West Jutland, University Hospital of Southern Denmark, Esbjerg, Denmark

**Keywords:** Cryoneurolysis, Osteoarthrities, Knee pain, Exercise

## Abstract

**Objective:**

Pain is the principal symptom in knee osteoarthritis (OA). Current non-operative treatment options have only moderate effects and often patients experience persistent pain or side-effects. Novel advances in the field of cryoneurolysis applies low temperatures to disrupt nerve signaling at the painful area, providing pain relief. The primary aim of this randomized controlled trial (RCT) is to investigate if cryoneurolysis is superior to sham at decreasing pain intensity 2 weeks after the intervention in patients with knee OA. Secondary aims are to explore effects on pain, quality of life and functional performance over 24 months.

**Methods:**

This two-arm, parallel-group RCT, approved by the Regional Ethics Committee, will randomly allocate patients (*n* = 94) to a cryoneurolysis intervention group + standardized education and exercise (CRYO) or a sham group + standardized education and exercise (SHAM) (1:1 ratio). Both groups will be assessed at baseline, 2 weeks post intervention, post education and exercise and at 6, 12 and 24 months after cryoneurolysis. The primary outcome is the NRS knee pain intensity score assessed 2 weeks post the intervention. Secondary outcome measures include functional performance (chair-stand test, 40 m walk, stair test and maximum voluntary contraction of the knee), patient reported outcomes (quality of life (EQ5D), Knee and osteoarthritis outcome scores (KOOS), among others), use of analgesics, and adverse events over 24 months.

**Impact statement:**

Cryoneurolysis could potentially provide an effective, safe and non-pharmacological therapeutic option to treat pain in OA patients. The potential benefits include increased functional capacity and quality of life as a result of significant pain relief and improved benefits of physical exercise.

**Trial registration:**

Clinicaltrials.gov, NCT03774121, registered 3 March 2018, http://www.clinicaltrials.gov

## Background

Chronic pain represents a major challenge worldwide, with significant clinical, social and economic implications. In Denmark and the rest of Europe, 20% of all chronic pain conditions is related to osteoarthritis (OA) [1]. There are over 300.000 people diagnosed with OA per year in Denmark alone, and the incidence of this pathology will increase significantly [2]. The pain and loss of function associated with OA, results in a considerable amount of years lived with disability [3] and has significant socioeconomic consequences, estimated at 1 - 2.5% of the gross domestic product in western countries [4]. Knee OA in particular, has a high prevalence rate compared to other types of OA, and is also present in the younger working age population [5].

The treatment of knee OA typically focuses on pain relief, however, the effects of current conservative treatment options remain small to moderate and most are associated with side effects [6]. In many cases, patients may be subjected to partial/total knee arthroplasty (TKA). TKA is considered to be an effective treatment for end-stage knee osteoarthritis [7], however, more than 20% of patients receiving TKA, experience persistent and unchanged pain post-surgery [8–10]. Therefore, effective and low-risk strategies are needed [11, 12]. Cryoneurolysis, which is the application of low temperatures [− 20 °C to − 100 °C] to a target percutaneous peripheral nerve, causing Wallerian degeneration [13, 14], disrupts nerve function while structural elements of the nerve bundle remain intact. This allows for complete regeneration and functional recovery of the nerve over time [15, 16] and has shown promising short and long-term results in a variety of chronic pain conditions such as lumbar facet joint pain [17], plantar fasciitis [18], occipital neuralgia [19], post thoracotomy pain syndrome [20], and Morton’s neuroma [21]. The ability to target the genicular nerves to reduce pain around the knee has been reported by studies applying radio frequency ablation (RF) [22–24] but cryoneurolysis has in that respect been associated with less adverse effects [25]. Radnovich et al. targeted the infrapatellar branch of the saphenous nerve (ISN) to reduce pain in patients with knee OA [26]. The authors reported significant pain relief for up to 150 days and no serious adverse effects. In another study, Dasa et al. introduced preoperative cryoneurolysis to the ISN and the anterior femoral cutaneous nerve (AFCN) prior to TKA in patients with knee OA and observed a statistically significant reduction in hospital stay (days), and a decrease in prescribed opioids [27]. These results provide clinical evidence, suggesting that cryoneurolysis treatment is a safe procedure, that may reduce both pain and symptoms in patients with knee OA [14, 28]. Further adequately powered prospective randomized controlled trials (RCTs) are needed to confirm the efficacy and safety of cryoneurolysis treatment in patients with knee OA.

In Denmark, a standardized education and neuromuscular exercise program (GLA:D) has been implemented in the national clinical guidelines by the Danish Health Authority, for the treatment of knee and hip OA in clinical practice [29]. Recent studies show that exercise reduce pain and improve function in people with knee or hip OA [30, 31]. Despite of these reports, the beneficial effects remains moderate with difficulties in maintaining these effects at long-term follow-up [29]. In addition the implementation of the exercise program is not optimal [12] and includes a significant discontinuation rate for patients reporting high pain levels [29]. In this line, pain and muscle weakness, among others, have been reported to be major barriers for physical exercise [32, 33]. The application of cryoneurolysis treatment as an effective pain reducing treatment prior to GLA:D might provide significant pain relief and improve patients’ ability to produce force, resulting in improved adherence, exercise effectiveness and long-term benefits [34]. Currently, no studies have reported the effects of cryoneurolysis treatment on pain and functional performance in conjunction with a standardized education and exercise programme (GLA:D), in patients with knee OA.

### Objective

The primary objective of this RCT is to investigate if cryoneurolysis is superior to sham at decreasing pain intensity 2 weeks after the intervention in patients with painful knee OA.

The secondary objective is to explore the safety and effectiveness of cryoneurolysis followed by GLA:D and to assess long term effects.

## Methods

This study protocol describes the design of an ongoing parallel-group RCT conducted at the Department of Neurology, University Hospital of Southern Denmark, Esbjerg, Denmark. The study protocol conforms to the Standard Protocol Items: Recommendations for Interventional Trials (SPIRIT) (see also appendix, Table 2), and The Consolidated Standards of Reporting Trials of Non-pharmacological Treatments (CONSORT NPT) will be used as a guideline for reporting this trial. The study is conducted according to the declaration of Helsinki and is approved by The Regional Committees on Health Research Ethics for Southern Denmark (S-20180089) and registered in ClinicalTrials.gov (NCT03774121).

### Participants and recruitment procedure

**Fig. 1:**
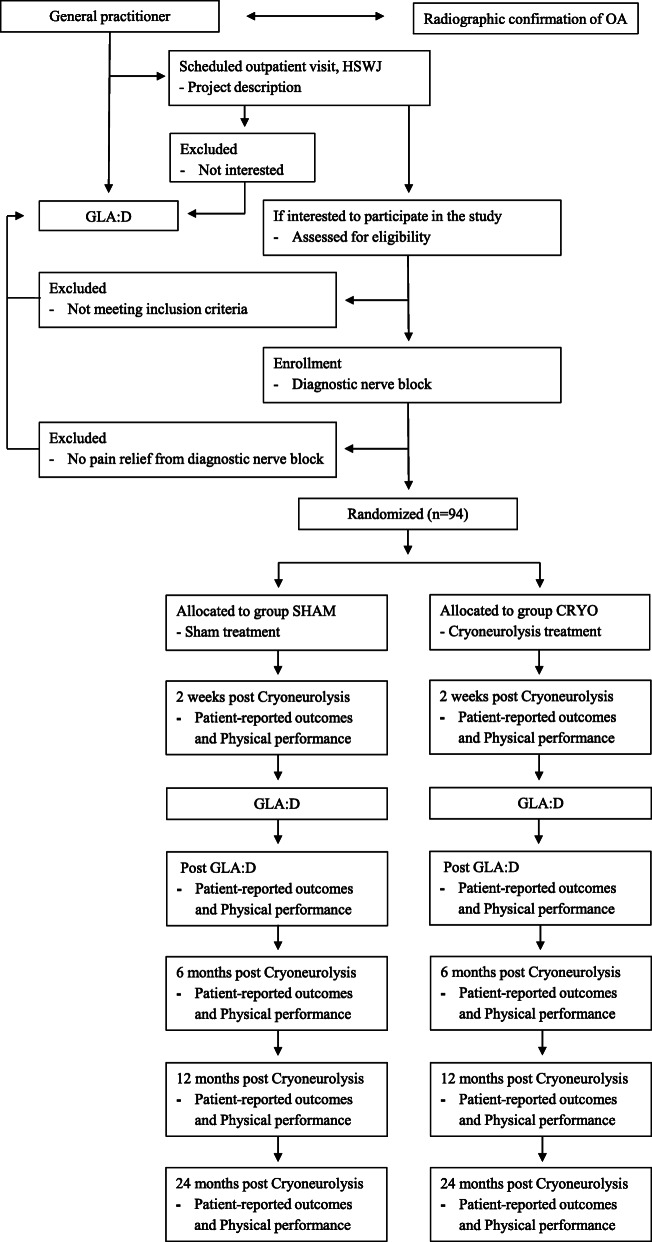
Flow chart

This study is recruiting patients with pain and knee OA that are referred to GLA:D by their general practitioner, prior to assessment of surgery eligibility at the hospital (Fig. 1).

Patients are screened according to the eligibility criteria outlined below, and the diagnosis is confirmed by further examination by an orthopaedic surgeon confirming knee OA and OA related pain. Eligible patients are invited to proceed to a second visit where nerves around the knee are identified using ultrasound (US) and electrical nerve stimulation followed by a diagnostic genicular nerve block. If patients experience a ≥ 50% decrease in knee pain intensity (NRS) as a result of the nerve block, they will be randomly allocated to either a cryoneurolysis intervention group (CRYO) or a sham group (SHAM).

#### Inclusion criteria

- Referred to GLA:D [29] by a physician.
- Age ≥ 18 years
- Chronic knee pain for a minimum duration of 6 months.
- Pain intensity ≥4 on the Numeric Rating Scale (NRS).
- Radiographic confirmation of osteoarthritis; Grade 2-4 changes according to the Kellgren-Lawrence classification system.
- A decrease of ≥50% in NRS scores with diagnostic genicular nerve block.
- Written and oral understanding of Danish.

#### Exclusion criteria

- History of systemic inflammatory conditions such as rheumatoid arthritis.
- Previous recipient of cryoneurolysis for the knee.
- Use of hyaluronic acid within the previous 30 days.
- Injection of corticosteroid within the previous 3 months.
- Clinically significant structural abnormities affecting locomotion and knee function aside from osteoarthritis and which might cause chronic knee pain.
- Body mass index ≤18 and ≥ 40 kg/m2.
- In treatment for other pain conditions.
- Pregnancy
- Coagulopathy
- Uncontrolled serious disease (cancer, diabetes, etc.)
- Disease associated with reactions to cold, such as cryoglobulinemia, cold urticarial and Renaud’s syndrome.

### Randomization

Randomization is performed as computer-generated block randomization with a 1:1 allocation ratio using random block sizes of 2, 4 and 6. The randomization restrictions will not be disclosed to ensure allocation concealment and the sequence will be performed by an external co-investigator. To account for the placebo effect and reduce the risk of bias, the patients, therapists and data-manager will be blinded to the allocation. Blinding will be assured using a sham trial that includes the same procedures as cryoneurolysis treatment but without any freezing temperatures.

The allocation code is concealed in a sealed envelope, that will be available for the surgeon performing the cryoneurolysis procedure only. In case of unexpected issues and if deemed absolutely necessary by the investigators and physician, unblinding will occur according to emergency unblinding procedures that will maintain the integrity and confidentiality of the study.

### Interventions

#### Active and sham cryoneurolysis

The interventions involve two visits.

##### First visit

Radiographic confirmation and clinical determination of knee OA as pain generator followed by the identification of the infrapatellar branch of the saphenous nerve (IBSN) and anterior femoral cutaneous nerve (AFCN). Predefined areas for each nerve are marked directly on the patient [35, 36]. The area is searched using a transcutaneous electrical nerve stimulation (TENS) wand. Areas where the patient reported a response are marked, and then retraced with incrementally lower current to further specify nerve location. The location is finally visually confirmed using ultra-sound. Nerve structures can be distinguished by its hypoechoic nerve fascicles among the hyperechoic epineurium forming a honeycomb-like structure in short-axis view. A diagnostic nerve block using Ropivacain (5 mg/ml) is performed at each location guided by ultra-sound. All patients reporting a decrease of ≥50% in NRS pain scores are scheduled for a second visit for cryoneurolysis.

##### Second visit

Ropivacain (5 mg/ml) is injected before treatment, locally at the insertion-point, 4-6 cm from target nerve locations. This allows for continuous patient feedback during the following procedures. The cryoneurolysis probe (Iceseed 1.5, Galil Medical Ltd.) is inserted in proximity of the target nerve, guided by ultrasound visualization to accurately determine the location of the nerve and to account for adjacent neurovascular structures and variations in anatomical structures. Cryoneurolysis are performed with a single freeze cycle; 30s at an effect of 20%, and 2 min 30s at 60% effect. After each freezing cycle, 1 min active thaw and 1 min passive thaw is used. The machine used for cryoneurolysis is a VisualICE, (Galil Medical Ltd.), which utilizes Argon as a coolant and Helium to thaw. This technology allows for *reversible* destruction of nerves, also known as Wallerian degeneration, that prevents nerve signaling and potentially alleviate pain and motor dysfunction in a number of medical conditions.

The sham intervention includes the same procedures as described above but using a sham probe that does not allow for any freezing temperatures. Thus, visible marks as a result of the procedures will be similar in both groups.

#### GLA:D

Following the cryoneurolysis intervention, both groups will participate in GLA:D [29] for a duration of 8 weeks provided by specialized physiotherapists. The GLA:D program consists of patient education and neuromuscular exercise. Patient education consists of three sessions over the course of 2 weeks given by trained physiotherapists and focus on giving the patient knowledge on osteoarthritis and treatment with exercise. Following patient education, patients participates in a 6-week group-based NEuroMuscular Exercise program (NEMEX) with a total of 12 sessions, each session lasting for 60 min [31]. Patients who do not wish to or are not able to participate in the supervised exercise program, have the choice to perform the program at home, with detailed instructions by the physiotherapist. The NEMEX of the GLA:D program is performed in groups and with the supervision of an experienced physiotherapist specialized in training of musculoskeletal disorders.

### Outcomes

The patients will be assessed at baseline, 2 weeks post intervention, after completion of GLA:D and 6, 12- and 24- months post cryoneurolysis. The tests will include both patient-reported outcomes (PRO) and objective functional performance tests. The effects of group CRYO and group SHAM will be compared at each timepoint (Table 1).

#### Primary outcome

The primary outcome is the change in patient reported knee pain intensity assessed using the numeric rating scale (NRS) from baseline to 2 weeks post cryoneurolysis compared between group CRYO and SHAM. NRS consists of an 11-point scale between 0 and 10-, anchored by two verbal descriptors, “no pain” for the score of 0 and “worst pain imaginable” for the score of 10. Respondents are asked to rate their pain intensity on average within the past 24 h. The NRS instrument has been validated in diverse populations and has been widely applied in clinical and research settings [37].

#### Secondary outcomes

Functional performance will be evaluated by the 30 s chair-stand test, the 40 m fast-paced walk test, the 9-step stair-climb test and isometric knee MVC force [38].

*The 30 s chair-stand test* [39] consists of repeated sit-to-stand movement for a duration of 30 s. The starting position is seated, with feet placed flat on the floor, shoulder width apart and with the arms crossed on the chest. The position change to standing, with hips and knees fully extended, followed by sitting back down, with bottom fully touching the seat. The test is performed with usual footwear and the chair should be with a straight back, with no arms, placed against a wall. In cases where the movement cannot be performed even once, the hands are allowed to be placed on the legs or a regular mobility aid can be used – the result is then reported as an adapted test score. The outcome is the total number of complete chair stands performed for the duration of the task (one chair stand represents a stand followed by a sit movement).

*The 40 m fast paced walk test* [40] consists of walking as fast as possible, but still safely, along a 10 m marked walkway, then turning around a cone / tape and return. This is then repeated for a total distance of 40 m. The test is performed with usual footwear and regular walking aid is allowed and recorded. The outcome is expressed as speed. i.e. walking distance (40 m) divided by the time to perform the task (s). Timing is paused during turns.

*The 9-step stair-climb test* [41] consists of the ascend and descend a flight of stairs as fast as possible, but still safely. The flight of stairs preferably has 9 steps, step height appx. 20 cm, with handrails. The test is performed with usual footwear and regular walking aid is allowed and recorded. The outcome is the total time to perform the task (s).

Quadriceps strength will be assessed measuring isometric *MVC force* of the knee extensors (Bofors Elektronik, Karlskoga, Sweden). Patients will be seated in a chair with knee and hip flexed at 90° and with the pelvis and chest restrained by straps. A non-extensile chain attached to the back of the chair and connected to a force transducer will be placed just proximal to the malleolus. The patient will then be asked to perform three knee extensions pushing as hard as possible against the chain, with 1 min rest in between. The highest peak value out of the three MVCs will be taken as the MVC force.

In addition to pain intensity, PRO-data will be collected using PainData, which is an electronic questionnaire system and database with multiple integrated questionnaires, including:

- Knee Injury and Osteoarthritis Outcome Score (KOOS). To assess patients’ opinion about their knee and associated problems [42].
- EQ-5D. To assess generic quality of life [43].
- Pain Catastrophizing Scale. To assess the extent of catastrophic thinking [44].
- Patient Health Questionaire (PHQ9). To assess the extent of depression [45].
- Generalized Anxiety Disorder (GAD7). To assess the extent of anxiety [46].
- Pain intensity (NRS) and location (electronic drawing on a 2D model).
- Self-reported use of analgesics.

Adverse events will also be reported and are defined as any undesirable experience during the trial leading to contact with the healthcare system (general practitioner, emergency room or hospital). All adverse effects will be assessed during follow-ups using both pre-specified symptom inventories and open-ended questions.

**Table 1:** Timeline of the study – outcomes and variables assessed during the trial period

Timepoint	Study period								
	Enrolment		Allocation	Post-allocation			Follow-up		
	-4w	-1w	0w	2w	10w	na	24w	48w	96w
Enrolment:									
Eligibility screening	X								
Informed consent	X								
Genicular nerve block		X							
Allocation			X						
Interventions:									
Cryoeurolysis (CRYO)			X						
Sham treatment (SHAM)			X						
GLA:D (SHAM & CRYO)				X	X				
Assessments:									
Pain			X	X		X	X	X	X
Functional performance			X	X		X	X	X	X
KOOS			X	X		X	X	X	X
EQ5D			X	X		X	X	X	X
Adverse effects			X	X		X	X	X	X
Analgesics			X	X		X	X	X	X

*w* Weeks, *na* Not applicable (GLA:D start time may vary)

### Sample size

The planned number of trial participants is based on the null-hypothesis, assuming no difference between cryoneurolysis treatment and sham. Estimating the sample size for a two-sample means test with a level of significance at 0.05, assuming a common standard deviation (SD) of 3 in NRS pain intensity scores indicates that for the intention-to-treat (ITT) population, 74 individuals is required to obtain a power of at least 80% to establish a minimal clinically important difference (MCID) of 2 in NRS pain scores [47]. The MCID and common standard deviation is based on previous findings with a similar patient group and intervention [48]. With an expected drop-out rate of 20%, a total of 94 individuals will be included in the project, 47 in each group.

### Statistical methods

To evaluate the distributions of the continuous outcomes, visual inspection of the studentized residuals will be applied to evaluate whether the assumption of normality is reasonable. Data will be reported as differences between group means (means ± standard deviations, 95% CIs) if normal distributed, otherwise as medians with interquartile ranges. Categorical data will be reported as numbers and proportions. An Intention-To-Treat (ITT) analysis will be used for all allocated patients. Mixed linear regression with the assumption of unstructured covariance will be used to model the effect of the cryoneurolysis treatment over time and to take into account the repeated measures by including individuals as random variables. Time, group of treatment (cryoneurolysis or SHAM) and the Group, time interaction will be used as fixed effects to estimate different patterns of change over time. The results will be illustrated using marginal effects that will be calculated using the margins command of STATA16. If the 95% CIs do not overlap, data will be considered statistically significant. A statistically significant difference of at least two in NRS scores between groups, will be interpreted as a MCID.

### Data monitoring

The current project will not require a formal data monitoring committee. This decision is based on the minimal known risks associated with the intervention and little expected disturbance to the clinical equipoise. Any adverse effects will be recorded and reported according to the guidelines to the Regional Committee on Health Research Ethics for Southern Denmark within 7 days. Once a year the lead investigator will report all expected and unexpected adverse effects that have occurred during that period, with an evaluation of patient safety.

The leading investigators will review the trial processes and data continuously. In addition, a status report will be sent for approval to the Danish Health Authority each year.

### Data management

All data will be kept electronically and filed according to a participant code. Data entry will always be handled by the same investigator, who will use unambiguous and standard terminology based on predefined study forms. Complete back-up of all data will be performed regularly and stored on OPEN’s servers in the Region of Southern Denmark, using Research Electronic Data Capture (REDCap). REDCap store data via an encrypted connection with restricted access and fulfil the demands for data security. Data will be stored for a duration of 10 years. The project will be reported to the Danish Data Protection Agency and will be handled according to the regulations of the Act on Processing of Personal Data. The full data set will be available to the lead investigators only.

### Ethics

The study is conducted according to the declaration of Helsinki and is approved by The Regional Committees on Health Research Ethics for Southern Denmark (S-20180089) and registered in ClinicalTrials.gov (NCT03774121). Given the study design, eligibility criteria and safety measures, the current project does not include special risks for the participating patients. The majority of risks does not require medical attention and serious adverse effects as a result of local anesthetics and cryoneurolysis are very rare. In contrast cryoneurolysis could potentially provide an effective, safe and minimally invasive option to treat pain in OA patients.

The inclusion of a sham group is necessary to test the hypothesis because of the large reported placebo effects in invasive interventions [49]. In addition, the associated risks are minimal and the nature of misleading, in regards to the administration of the sham trial, will be adequately disclosed and accepted by the patient during the informed consent process. The sham group is implemented according to current standards [50].

## Discussion

### Impact and significance of the study

Current pain-relieving therapies show small to moderate effects and a significant portion of patients who receive surgery (TKA) report unchanged pain intensity levels post-surgery. This indicates that there is a need for additional therapies that could supplement or improve the existing therapies. A significant difference in the change in pain intensity between sham and intervention post cryoneurolysis treatment would indicate that cryoneurolysis is an effective non-pharmacological therapeutic option to treat pain in OA patients. Outcome measures include both pain, social and psychological factors and functional capacity, which allows for a thorough understanding of treatment effects. The novel addition of GLA:D in combination with cryoneurolysis will elucidate whether feasibility and effectiveness of exercise increase if pain is attenuated and if the potential pain-relieving effects of cryoneurolysis can be extended. The potential benefits include increased functional capacity and quality of life as a result of significant pain relief and improved benefits of physical exercise, ultimately postponing or making surgical intervention unnecessary. This could have a significant impact on patients’ lives as well as significant socioeconomic consequences and could change the clinical landscape in the treatment of OA. Finally, an important perspective, is the application of cryoneurolysis in other areas – such as managing pain, related to surgery, to improve rehabilitation.

If the results indicate that cryoneurolysis does not elicit additional pain relief when compared to sham, it might indicate that cryoneurolysis treatment is not suitable for the treatment of OA related pain or at least not in the way it is applied in the current study.

Importantly, the freezing protocol in this study results in reversible nerve damage, allowing for the reinnervation of the sensory receptors over time [15, 16]. It also means that pain sensation might return over time [17, 26, 51, 52]. To maintain long-term pain-relieving effects, this study is the first to combine cryoneurolysis with a standardized education and exercise program. The analgesic effects of exercise are well established, however, the effects remain moderate and difficult to maintain over time [29]. Cryoneurolysis of the genicular nerves allows for potential long-term pain relief (> 3 months), without affecting motor control, which could help exercise feasibility and effectiveness - providing long-term benefits of both cryoneurolysis and exercise [34]. In the current study, patients are referred to GLA:D by their general practitioner and is performed independently of the study at specialized physiotherapists, post cryoneurolysis treatment. Differences in practice and starting time might occur between patients depending on the physiotherapist and some may choose to perform parts of their training at home. These factors might confound the outcome of the exercise program but is consistent with clinical practice. The primary investigator facilitates GLA:D participation.

### Strength and limitations

This study is a blinded randomized controlled sham trial that includes long-term follow ups allowing for an evaluation of the long-term effects of treatment. The sham intervention includes the same procedures as in cryoneurolysis treatment, emulating both physical marks and sensory experiences such as sound and vision. Despite of this, some performance bias cannot be excluded, and some attrition bias might occur, due to patients seeking treatment elsewhere – expectedly from the sham group. The inclusion of a sham group is necessary to test the hypothesis because of the large reported placebo effects in OA trials [49]. Treatment with cryoneurolysis is still in its early stage and further studies are needed to determine methodological strategies optimizing its potential therapeutic effects. To achieve long-term pain relief, significant degeneration of the sensory nerves is required. The extent of nerve damage depends on several factors including temperature, contact area, freezing rate, exposure time, thawing strategy and cell type. The current study applies a conservative freezing protocol, that is relatively short ~ 3 min, a single freezing cycle and not at full effect (slower freezing). This might reduce potential risks associated with the procedure but might also attenuate treatment effects. Other studies use up to several cycles and freezing periods up to 10 min, which might induce pain relief for a longer duration due to increased nerve degeneration. These studies, however, tend to focus more on tissue destruction rather than degeneration. Further studies are needed to determine optimal freezing protocols relative to different use cases. The target nerves, the identification of the target nerves and placement of the cryo probe is equally as important and might have a significant impact on the results. The temperature gradient away from the probe increases drastically [4, 7]. The typical isotherm with the needle (IceSeed 1.5, Galil Medical) used in this study has a temperature of − 40 °C in an area equivalent to 11x20mm. Already at 20x27mm the temperature has risen to − 20 °C and at 33x38mm the temperature is up to 0 °C [8]. Thus, to attain temperatures necessary for a relevant impact on sensory nerves for long-term pain relief [− 20 °C - 100 °C], probe placement relative to the nerve is crucial. The current study uses a combination of anatomical landmarks, electrical nerve stimulation and ultrasound to accurately determine nerve locations and to account for variation in surrounding anatomical structures. This both increase precision and safety of the procedure. Nevertheless, it is in some cases difficult to identify and differentiate nerve structures with ultrasound which might cause variable results. The procedure is in this study performed by the same surgeon throughout, trained to identify neural structures and to perform cryoneurolysis. In addition, local anesthesia is used locally only at the injection site, 4-6 cm from the ‘target’ nerve. This allows for continuous feedback on the effect of treatment.

### Dissemination of results

The obtained results will be made publicly available within 1 year after the end of the project. This will include publication of the obtained results in international scientific peer-reviewed journals - adhering to the recommendations of the Vancouver convention. Published papers will have open access to ensure a broad distribution and the results will be presented at international scientific conferences. The results will also be presented for the general public and distributed across public platforms (e.g. regional and national press, internet sites, etc.) if copyright allows.

## Data Availability

Not applicable.

## Appendix

**Table 2:** WHO data registration items

**Data category**	**Information**
Protocol version	Version 1 - 21 January 2021
Primary Registry	Clinicaltrial.gov
Date of Registration	12-03-2018
Sources of Monetary	Danish Health Authority, Danish Rheumatism Association
Primary Sponsor	Department of Neurology, Hospital South West Jutland, Esbjerg Denmark
Secondary Sponsor	Research Unit of Health Sciences, Hospital South West Jutland, Esbjerg Denmark
Contact for Public Queries	Niels-Peter Broechner Nygaard
Contact for Scientific Queries	Niels-Peter Broechner Nygaard
Country of Recruitment	Denmark
Health Condition	Osteoarthritis (DM17)
Interventions	Cryoneurolysis
	Sham
	GLA:D
Key inclusion and exclusion criteria	Inclusion: Referred to GLA:D, Chronic pain > 6 months, Pain intensity ≥40 mm on the NRS pain scale, OA confirmed by radiography, 50% reduction of pain after diagnostic nerve block
	Exclusion: Systemic inflammatory disease, In treatment for other pain conditions, uncontrolled serious disease (cancer, diabetes, etc.)
Study type	Interventional
	Allocation; Randomized controlled; Parallel assignment; Single-blinded (patients and data-manager)
	Primary purpose; Safety and effectiveness
Date if first enrollment	26-06-20,189
Sample size	*n* = 94
Recruitment status	Recruiting
Primary outcome	Change in Pain intensity score (NRS) after cryoneurolysis, compared between intervention and sham
Key secondary outcomes	Functional performance
	Quality of Life
	Adverse effects
